# Machine Learning Multimodal Model for Delirium Risk Stratification

**DOI:** 10.1001/jamanetworkopen.2025.8874

**Published:** 2025-05-07

**Authors:** Joseph I. Friedman, Prathamesh Parchure, Fu-Yuan Cheng, Weijia Fu, Satyanarayana Cheertirala, Prem Timsina, Ganesh Raut, Katherine Reina, Josiane Joseph-Jimerson, Madhu Mazumdar, Robert Freeman, David L. Reich, Arash Kia

**Affiliations:** 1 Department of Psychiatry, Icahn School of Medicine at Mount Sinai, New York, New York; 2 Department of Neuroscience, Icahn School of Medicine at Mount Sinai, New York, New York; 3 Institute for Healthcare Delivery Science, Icahn School of Medicine at Mount Sinai, New York, New York; 4 Nursing Administration, Mount Sinai Morningside Hospital, New York, New York; 5 Department of Nursing, The Mount Sinai Hospital, New York, New York; 6 Department of Population Health Science and Policy, Icahn School of Medicine at Mount Sinai, New York, New York; 7 Department of Anesthesiology, Perioperative, and Pain Medicine, Icahn School of Medicine at Mount Sinai, New York, New York

## Abstract

**Importance:**

Automating the identification of risk for developing hospital delirium with models that use machine learning (ML) could facilitate more rapid prevention, identification, and treatment of delirium. However, there are very few reports on the performance of ML models for delirium risk stratification in live clinical practice.

**Objective:**

To report on development, operationalization, and validation of a multimodal ML model for delirium risk stratification in live clinical practice and its associations with workflow and clinical outcomes.

**Design, Setting, and Participants:**

This quality improvement study developed an ML model supported by automated electronic medical records to stratify the risk of non–intensive care unit delirium in live clinical practice using the Confusion Assessment Method as the diagnostic reference standard, with an iterative model update method. Data from patients aged at least 60 years admitted to non–intensive care units at Mount Sinai Hospital between January 2016 and January 2020 were used to train and test the ML model presented. The model was validated in live clinical practice from March 2023 to March 2024. Analysis of the model’s associations with workflow and clinical outcomes was conducted retrospectively in 2024, comparing hospitalized patients prior to deployment of any model version (pre-ML cohort) and during model clinical deployment (post-ML cohort).

**Main Outcomes and Measures:**

Outcomes of interest were area under the receiver operating characteristic curve, monthly delirium detection rates, median length of hospital stay, and daily doses of opiate, benzodiazepine, and antipsychotic medications administered.

**Results:**

The overall sample included 32 284 inpatient admissions (mean [SD] age, 73.56 (9.67) years, 15 157 [46.9%] women). A total of 25 261 inpatient admissions of older patients with both medical and surgical primary diagnoses represented the combined model testing and training cohort (median age, 73.37 [66.42-81.36] years) and live clinical deployment validation cohort (median [IQR] age, 72.11 [62.26-78.97] years), while 7023 inpatient admissions of older patients with both medical and surgical primary diagnoses represented the combined pre-ML (median [IQR] age, 74.00 [68.00-81.00] years) and post-ML (median [IQR] age, 75.33 [68.34-82.91] years) cohorts. The model presented is a fusion of electronic medical record patient data features and clinical note features processed by natural language processing. The results of model validation in live clinical practice included an area under the curve of 0.94 (95% CI, 0.93-0.95). Median (IQR) monthly delirium detection rates of inpatients assessed for delirium with the Confusion Assessment Method increased from 4.42% (95% CI, 3.70%-5.14%) in the pre-ML cohort to 17.17% (95% CI, 15.54%-18.80%) in the post-ML cohort (*P* < .001). Post-ML vs pre-ML cohorts received lower daily doses of benzodiazepines (median [IQR] 0.93 [0.42-2.28] diazepam dose equivalents vs 1.60 [0.66-4.27] diazepam dose equivalents; *P* < .001) and olanzapine (median [IQR], 1.09 [0.38-2.46] mg vs 2.50 [1.17-6.65] mg; *P* < .001).

**Conclusions and Relevance:**

This quality improvement study demonstrates the feasibility of a novel multimodal ML model to automate delirium risk stratification in live clinical practice. The model demonstrated acceptable performance in live clinical practice and may facilitate resource allocation to enhance delirium identification and care.

## Key Points

**Question:**

Can a machine learning model be used to accurately stratify risk of hospital delirium in live clinical practice?

**Findings:**

This quality improvement study including 32 284 inpatient admissions developed an automated multimodal machine learning delirium risk stratification model that demonstrated acceptable discriminative performance in live clinical practice. Additional analyses using 7023 admissions assessed for delirium with the Confusion Assessment Method showed that model deployment was associated with a significant 4-fold increase in delirium detection rates and significant reductions in daily doses of benzodiazepine and antipsychotic medications.

**Meaning:**

These findings suggest that a machine learning model may be used to automate delirium risk stratification in live clinical practice and may enhance delirium identification and care.

## Teaser

This quality improvement study describes the development, operationalization, and validation of a multimodal machine learning–based model for delirium risk stratification in clinical practice and its associations with workflow and clinical outcomes.

## Introduction

Delirium is a frequently occurring neuropsychiatric syndrome in hospitalized individuals, precipitated by various medical, surgical, pharmacological, and environmental factors. It manifests as acute mental status changes and is associated with short-term and long-term negative consequences, including increased morbidity, mortality, hospital readmission rates, functional decline, and extended hospital stays.^1,2^ Despite its frequent occurrence and negative consequences, the diagnosis of delirium is often missed and delayed in the hospital setting. Therefore, more timely diagnosis of delirium and accurate assessment of delirium risk followed by rapid treatment of existing delirium and preventative measures in patients with high risk are essential to mitigating these adverse outcomes.

To facilitate the more timely diagnosis of delirium in the hospital setting, there has been an increasing interest in applying artificial intelligence (AI) to risk stratification for the development of delirium in hospitalized patients. Consequently, there is a rapidly evolving body of research exploring the development of prediction models using machine learning (ML) and supported by electronic medical record (EMR) data for delirium that develops in hospital settings outside the intensive care unit (ICU).^3,4,5,6,7,8,9,10,11,12,13,14,15,16,17,18,19,20,21,22,23,24,25,26,27,28,29,30,31,32,33,34,35,36,37,38,39,40,41,42,43,44,45,46,47,48^ However, most of these models have not been tested in live clinical settings, resulting in a paucity of evidence for AI’s added value to workflow and clinical outcomes associated with delirium in the hospitalized patient.

More recently, a few publications of ML-based delirium risk models have investigated the benefit of using natural language processing (NLP) features in the model being evaluated.^11,25,32,33,46^ Furthermore, 2 publications have reported on the fusion of the traditional EMR-based features with NLP features in their ML-based delirium risk stratification model, with both supporting the model-boosting efficacy of the NLP component.^11,32^

Despite these advances, to our knowledge, only 4 publications have reported on performance of ML-based delirium prediction models in live clinical practice, with 3 reports testing the same model (without NLP).^9,22,48^ Two of these reports compared nursing- and physician-derived delirium risk assignment vs ML-based delirium risk assignment,^9,22^ and 1 study tested model predictive performance against a structured delirium assessment tool.^48^ Clinical outcomes were reported in only 1 of these studies: user acceptance, as assessed by an author-developed questionnaire.^22^ The fourth report described clinical deployment of ML-based prediction models (with an NLP component) for acute kidney injury, sepsis, and delirium, with delirium diagnosis based on EMR-derived diagnostic codes assigned at discharge and no report of clinical impact.^25^

To address these shortcomings, in this study, we aimed to report on the development, training, validation, and operationalization of a multimodal ML-based model for delirium risk stratification in live clinical practice, with sequential model optimization, using a vertical integration approach^49^ and present the results of the optimized model’s performance in live clinical practice and its associations with clinical workflow and clinical outcomes.

## Methods

### Study Design and Setting

This quality improvement study was approved by the Mount Sinai Hospital (MSH) institutional review board, which also granted a waiver of informed consent due to minimal risk based on the protected health information that was accessed. We followed the Standards for Quality Improvement Reporting Excellence (SQUIRE) reporting guideline. This study was conducted at MSH from July 2019 through March 2024 using an interrupted time-series analysis^50^ with a focus on evaluating the association of the EMR-supported ML-based delirium risk stratification model we developed with workflow and clinical outcomes.

### Model Development

Since 2016, a novel delirium intervention program has been operational within the MSH system, targeting patients who have already developed delirium. This program uses a modified smaller team compared with traditional multicomponent delirium prevention programs.^51^ The team is supported by extensive workflow automation, facilitated by custom tools using data connectivity, real-time monitoring, and automated multistep processes integrated into our EMR.^51^

Trained team members certified as reliable assessors^51^ use a digitized version of the Confusion Assessment Method (CAM),^52^ integrated into our EMR, to enter their patient assessments. These assessments are scored by an EMR-based smart tool that uses automated multistep processes to generate a diagnosis of delirium based on the preprogrammed criteria outlined in the CAM Manual and Training Guide.^53^ On a diagnosis of delirium, additional delirium smart tools are activated, and team members initiate treatment of identified patients.^51^

All patients aged 60 years and older admitted to non-ICU medical and surgical units who met the inclusion criteria^51^ received a CAM assessment by the delirium service prior to deployment of any version of our ML-based delirium risk stratification model. Due to the high volume of assessments and limited resources, patients received only a single CAM assessment during their hospitalization. To address these inefficiencies, the delirium service collaborated with the clinical data science team to develop and deploy an EMR-supported ML model to stratify risk of developing delirium in our hospital.

We used a vertical integration approach for the development, training, testing, and deployment training of our ML-based delirium risk stratification model as opposed to a model-centric approach, outlined by Zhang and colleagues.^49^ Briefly, work on model design and deployment at all stages was carried out by a cross-disciplinary team that considered whether the existing infrastructure worked synergistically and supported the model during development, testing, and deployment, with ongoing evaluation of clinical impact, user and other stakeholder feedback, and model upgrades responsive to all these elements.^49^ Consequently, 3 successive versions of the delirium risk stratification model were developed, tested, and deployed (eFigure 1 in Supplement 1). The currently deployed model at MSH presented here represents a fusion of optimized model versions leveraging both EMR patient data features and NLP-processed clinical note features, henceforth referred to as the fusion multimodal with NLP model.

### Cohorts

Data for fusion multimodal with NLP model training, testing, postdeployment validation, and analyses of model deployment associations with workflow and clinical outcomes were derived from visit-level EMR data from patients aged at least 60 years admitted or transferred to MSH non-ICU units from January 2016 to January 2020 and March 2023 to March 2024. We used 4 cohorts for these analyses. The fusion model training/testing cohort included inpatient admissions from January 2016 to January 2020 with at least 1 CAM assessment performed. The fusion model live clinical deployment validation cohort included inpatient admissions assessed by the fusion model from March 1, 2023, to March 31, 2024. The pre-ML cohort included inpatient admissions with at least 1 CAM assessment performed during a 13-month period before model deployment (March 1, 2018, to March 31, 2019) to assess workflow and clinical outcomes before any model version was deployed. Finally, the post-ML cohort included inpatient admissions with at least 1 CAM assessment performed during a 13-month period after model deployment (March 1, 2023, to March 31, 2024) to assess workflow and clinical outcomes during the model’s live clinical deployment. The pre-ML and post-ML cohorts were created to ensure that comparison of workflow and clinical outcomes was conducted on groups derived from equal periods of time.

### Model Development Data Sources

Visit-level data from patients aged 60 years and older admitted or transferred to MSH non-ICU units between January 2016 and January 2020 were sourced from multiple data feeds: the Admission-Discharge-Transfer platform provided demographic and admission data, structured clinical assessments (including laboratory results and vital signs) were extracted from our EMR software (Epic; Epic Systems), electrocardiogram measurements were obtained from MUSE version 9 (GE HealthCare Technologies), and unstructured clinical data (eg, progress notes and care notes) were extracted from the EMR.

### Training Label

Patients were classified as either delirium-positive or delirium-negative based on standardized scoring of the CAM.^53^ The time of delirium onset (*t*_0_) was defined as the timestamp of the first positive CAM assessment indicating delirium, or the timestamp of the final negative CAM assessment.

### Structured and Semistructured EMR Data Processing

The model’s input relies on clinical observations. To standardize input data, a sampling module was developed to apply adaptive logic, ensuring a fixed number of observations within predefined intervals. This approach creates consistent, reproducible observation arrays for each type, accommodating diverse clinical scenarios.^54^ A time series was constructed by specifying a sampling window and frequency relative to the risk stratification time (*t*_p_). The sampling window was determined based on variable availability, optimizing data completeness and minimizing missing values (eFigure 2 in Supplement 1). The risk stratification time (*t*_p_) was set to 24 hours prior to the CAM assessment, while the sampling frequency defined the standard intervals between clinical measurements, ensuring consistency across observations. To handle the missing values in the numerical variables, the across-cohort median value was imputed for each variable. For each categorical variable, a specific encoding map was used to handle missing values. For example, the encoding map for gender was: men: (0, 0, 1), women: (0, 1, 0), unknown: (1, 0, 0), and missing value: (0, 0, 0). Patient race and ethnicity were identified in accordance with our EMR’s documentation workflow and recorded by nursing staff at admission. Race and ethnicity were categorized as Asian, Black or African American, Hispanic, White, and other (eg, American Indian or Alaska Native, Native Hawaiian or Pacific Islander, multiple races, and other). For each variable, an array of sampled observations was constructed and subsequently assembled into a feature vector.

### Clinical Note Preprocessing

For each inpatient admission included in the fusion model training and testing cohort, clinical notes, including care notes (submitted by registered nurses) and progress notes (submitted by residents, fellows, attending physicians, nurse practitioners, physician assistants), were aggregated into a text corpus. This corpus encompassed care and progress notes sampled from the 12-hour window preceding the risk stratification time (eFigure 3 in Supplement 1). The text corpus was processed using a sentence detection module to segment the text into individual sentences, which were then input into an NLP pipeline for tokenization, stemming, lemmatization, and the creation of 1-g and 2-g bag-of-words models. Term frequency rate was calculated at the encounter level across the cohort. Words with a term frequency rate of at least 0.3 in notes of patients classified as delirium-positive were selected as candidate features. The resulting feature list was categorized into 3 primary categories: diagnoses, signs and symptoms, and medications. Expert clinical feedback was used to refine the feature selection, focusing on those with relevance to the presentation of delirium. These selected features were then assembled into a feature vector.

### Clinical Note Classifier Development

The historical cohort dataset was randomly split into training (70%) and testing (30%) subsets. Due to the significant class imbalance (95% delirium-negative vs 5% delirium-positive), random undersampling was applied to the training set to achieve a balanced distribution of 50% negative and 50% positive. Visits without clinical notes were excluded from the training set. A 10-fold cross-validation was used to train the model using the random forest algorithm from the open-source Apache Spark project ML library,^55^ and recursive feature elimination was used for feature selection. After hyperparameter tuning, recursive feature elimination was implemented to reduce the number of features. For feature elimination, we applied an area under the receiver operating characteristic curve (AUROC) score threshold of 2.5% or less to permanently remove features with minimal contribution.

### Fusion Multimodal With NLP Model Development

To develop the fusion multimodal with NLP model, the undersampled training set was used, incorporating feature vectors derived from both structured and semistructured observational data. The NLP risk score was appended to these feature vectors. A 10-fold cross-validation procedure was used to train the model using the random forest algorithm. Following hyperparameter optimization, the recursive feature elimination method was applied to reduce the number of features (eFigure 4 in Supplement 1).

### Fusion Multimodal With NLP Model Deployment in Live Clinical Practice

Since the live clinical deployment of the fusion model on February 27, 2023, delirium risk stratification at MSH has been conducted daily for every patient aged at least 60 years admitted or transferred to non-ICU medical and surgical units. The resulting delirium risk value is visualized in our EMR’s patient lists with color coding, with peach indicating high risk (risk ≥0.55) and green, low risk (risk <0.55). Hovering over these risk values opens a popup providing specific features of the model-based risk prediction. MSH delirium service assessors use this risk visualization to prioritize CAM screening of patients at high risk for delirium. Once the CAM assessments are completed and documented in the digitized version of the CAM in our EMR,^51^ these patients are suppressed from the prediction model for 5 days. Subsequently, they are once again risk stratified by the model. Model use and performance are tracked prospectively using a real-time dashboard.

### Statistical Analyses

Differences in demographic and clinical characteristics between pre-ML and post-ML cohorts were analyzed using median values (due to significant skewness), with the Kruskal-Wallis test for continuous variables and the χ^2^ test for categorical variables. Elixhauser Comorbidity Index was calculated for all patients based on all secondary *International Statistical Classification of Diseases, Tenth Revision, Clinical Modification* (*ICD-10-CM*) diagnoses.^56^

#### Model Assessment

Sensitivity (recall), specificity, F1 score (how good the model is at identifying patients with and without the diagnosis), and the AUROC were calculated with 95% CIs using the scikit-learn library and custom Python scripts^57^ to evaluate and compare the discriminatory performance of the different models developed during fine-tuning and during clinical deployment of the fusion model.

#### Workflow and Clinical Outcomes

Workflow changes were analyzed by comparing monthly delirium detection rates, calculated as the ratio of CAM-positive delirium screening results to total CAM assessments performed each month. Delirium detection rates were compared using logistic regression adjusted for age, sex, race and ethnicity, surgical or medical primary diagnosis, dementia present at assessment, and Elixhauser Comorbidity Index. Length of stay (LOS) in the hospital (in days) and quantities of medication administration were chosen as the clinical outcomes, as previously reported in association with our delirium service deployment.^51^ Opiate and benzodiazepine medications were specifically chosen due to their known delirium-inducing effects^58^ and antipsychotic medications were chosen because of the Food and Drug Administration’s black box warning about increased death risk in patients aged 65 years and older with dementia.^59^ Opiate medication administration was analyzed by comparing the proportion of patients receiving these medications and then comparing the intravenous morphine dose equivalents administered in milligrams per hospital day. Benzodiazepine medications administration were similarly compared using the diazepam dose equivalents administered in milligrams per hospital day. Similar analyses were conducted for the administration of the antipsychotic medications haloperidol, risperidone, olanzapine, and quetiapine. Given the highly skewed distribution of the selected clinical outcomes, we applied the probabilistic index model to compare the LOS and medication administrations, adjusting for the same covariates. *P* values were 2-sided, and statistical significance was set at *P* < .05. Data were analyzed using R software version 4.3.3 (R Project for Statistical Computing).

## Results

The overall sample included 32 284 inpatient admissions (mean [SD] age, 73.56 (9.67) years, 15 157 [46.9%] women). A total of 25 261 inpatient admissions of older patients with both medical and surgical primary diagnoses represented the combined model testing and training cohort (median age, 73.37 [66.42-81.36] years) and live clinical deployment validation cohort (median [IQR] age, 72.11 [62.26-78.97] years), while 7023 inpatient admissions of older patients with both medical and surgical primary diagnoses represented the combined pre-ML (median [IQR] age, 74.00 [68.00-81.00] years) and post-ML (median [IQR] age, 75.33 [68.34-82.91] years) cohorts.

A total of 3992 inpatient admissions with at least 1 CAM assessment in the pre-ML cohort and 3031 admissions in the post-ML cohort were used to analyze the workflow and clinical outcomes. Differences of note in the pre-ML vs post-ML cohorts included higher proportions of Asian (260 patients [6.5%] vs 86 patients [2.8%]) and White (1758 patients [44.0%] vs 974 patients [32.1%]) patients; lower proportions of Black or African American patients (755 patients [18.9%] vs 789 patients [26.X%]), Hispanic patients (818 patients [20.6%] vs 827 patients [27.3%]), and patients with other race and ethnicity (342 patients [8.5%] vs 264 patients [8.7%]); a higher proportion of surgical patients (2045 patients [51.2%] vs 1179 patients [38.9%]); a lower Elixhauser Comorbidity Index (median [IQR], 15 [3-27] vs 24 [12-38]); and a lower LOS (median [IQR], 6.86 [4.07-13.13] days vs 13.11 [7.65-22.49] days) (Table 1). The demographic and clinical data for the fusion model training and testing cohort (5646 patients) and fusion model live clinical deployment validation cohort (19 615 patients), used to analyze model performance, did not show such differences and are shown the eTable in Supplement 1.

**Table 1.** Clinical and Demographic Characteristics of the Cohorts Before and After Deployment of the Multimodal With Natural Language Processing Delirium Prediction Model

Characteristic	Admissions, No. (%)		*P* value
	Predeployment^a^	Postdeployment^b^	
Admissions, No.	3992	3031	NA
Unique patients, No.	3587	2605	NA
Admissions with ≥1 CAM assessment, No.	3992	3031	NA
Delirium prevalence	184 (4.6)	519 (17.1)	<.001
Age, median (IQR), y	74.00 (68.00-81.00)	75.33 (68.34-82.91)	<.001
Gender			
Women	1933 (48.4)	1516 (50.0)	<.001
Men	2059 (51.6)	1492 (49.2)	
Missing	0	23 (0.8)	
Race and ethnicity			
Asian	260 (6.5)	86 (2.8)	<.001
Black or African American	755 (18.9)	789 (26.0)	
Hispanic	828 (20.6)	827 (27.3)	
White	1758 (44.0)	974 (32.1)	
Other^c^	342 (8.5)	264 (8.7)	
Unknown	69 (1.5)	68 (2.2)	
Missing	0 (0.0)	23 (0.8)	
Surgical or medical primary diagnosis			
Medical	1944 (48.4)	1816 (59.9)	<.001
Surgical	2045 (51.2)	1179 (38.9)	
Missing	3 (0.1)	36 (1.2)	
Dementia present at assessment			
Yes	449 (11.3)	325 (10.7)	<.001
No	3540 (88.6)	2670 (88.1)	
Missing	3 (0.1)	36 (1.2)	
Elixhauser Comorbidity Index, median (IQR)	15.00 (3.00-27.00)	24.00 (12.00-38.00)	<.001
Length of stay, median (IQR), d	6.78 (3.85-11.66)	13.11 (7.65-22.49)	<.001

Abbreviations: CAM, Confusion Assessment Method; NA, not applicable.

^a^ March 1, 2018, to March 31, 2019.

^b^ March 1, 2023, to March 31, 2024.

^c^ Other contains races including American Indian or Alaska Native, Native Hawaiian or Pacific Islander, multiracial, and other.

### Model Performance

**Figure 1.**
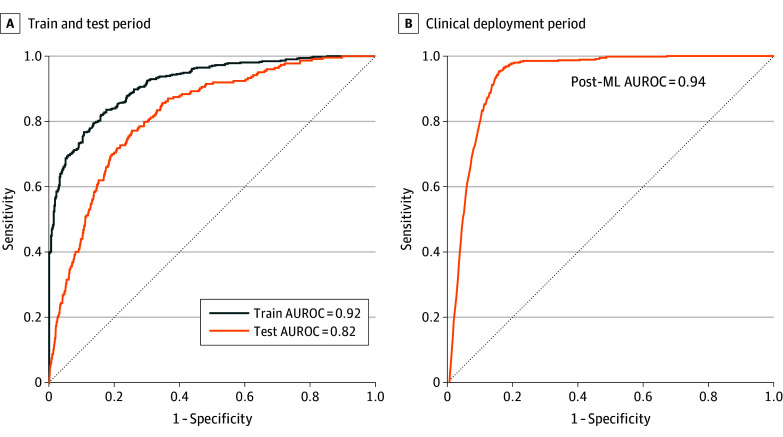
Receiver Operating Characteristic Curves for the Fusion Multimodal With Natural Language Processing Model to Predict Delirium AUROC indicates area under the curve receiver operating characteristic curve; post-ML indicates the period after model deployment.

Table 2 presents the discriminative performance statistics of the fusion multimodal with NLP ML-based delirium risk stratification model during the training, testing, and live clinical deployment periods. Figure 1 shows the corresponding AUROC curves. During live clinical deployment, the model achieved an AUROC of 0.94 (95% CI, 0.93-0.95), and at an operational risk probability threshold of 0.55, the model achieved a sensitivity of 83% (95% CI, 79%-87%), specificity of 90% (95% CI, 89%-91%), and an F1 score of 0.31 (95% CI, 0.28-0.34).

**Table 2.** Performance of the Fusion Multimodal With Natural Language Processing Delirium Risk Prediction Model During Training, Testing, and Live Clinical Deployment Periods

Period	Time period	Threshold	Total admissions, No.	Estimate (95% CI						
				Sensitivity	Specificity	PPV	NPV	Accuracy	F1 Score	AUROC
Training	January 2016 to January 2020	0.55	1008	0.82 (0.78-0.87)	0.83 (0.79-0.88)	0.84 (0.79-0.88)	0.82 (0.77-0.87)	0.83 (0.80-0.86)	0.83 (0.79-0.86)	0.92 (0.89-0.94)
Testing	January 2016 to January 2021	0.55	4638	0.75 (0.67-0.83)	0.76 (0.74-0.78)	0.14 (0.11-0.16)	0.98 (0.98-0.99)	0.76 (0.74-0.78)	0.23 (0.19-0.27)	0.82 (0.78-0.86)
Validation during fusion model live clinical deployment	March 1, 2023, to March 31, 2024	0.55	19 615	0.83 (0.79-0.87)	0.90 (0.89-0.91)	0.19 (0.17-0.21)	0.99 (0.99-1.00)	0.90 (0.89-0.90)	0.31 (0.28-0.34)	0.94 (0.93-0.95)

Abbreviations: AUROC, area under the curve receiver operating characteristic curve; NPV, negative predictive value; PPV, positive predictive value.

### Model Features and Their Importance

The top 20 variables ranked by Gini importance are summarized for the NLP model and fusion multimodal with NLP model in eFigure 5 in Supplement 1. The accuracy of the patients’ orientation to person, time, and place was identified as the strongest variable in the fusion model, closely followed by the composite NLP prediction score.

### Workflow Change

**Figure 2.**
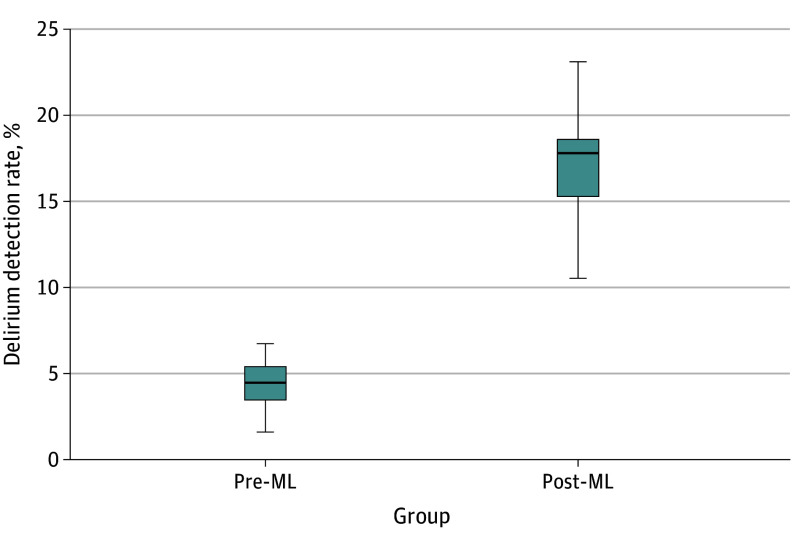
Comparison of Delirium Detection Rates Box-and-whisker plot comparing 5 summary statistics for the monthly delirium detection rates before any machine learning (ML) model deployment and following deployment of the multimodal with natural language processing ML-based delirium risk prediction model in live clinical practice. Delirium detection rates were calculated by dividing the number of positive Confusion Assessment Method (CAM) delirium screening results by the number of total CAM assessments each month. Whiskers indicate range; boxes, IQR; bold line, median.

Monthly delirium detection rates significantly increased during the deployment period. Median (IQR) rates increased from 4.42% (3.70%-5.14%) to 17.17% (15.54%-18.80%) (*P* < .001) (Figure 2).

### Clinical Outcomes

LOS was significantly higher in the post-ML cohort vs pre-ML cohort (median [IQR] LOS, 13.11 [7.65-22.49] days vs 6.78 [3.85-11.66] days). Compared with the pre-ML cohort, higher proportions of patients in the post-ML cohort received opiates (1352 patients [44.6%] vs 852 patients [21.3%]; *P* < .001) and olanzapine (31 patients [0.8%] vs 162 patients [5.3%]; *P* < .001). However, patients receiving these medications in the post-ML vs pre-ML cohorts received significantly lower daily doses of benzodiazepines (median [IQR] dosage, 0.93 [0.42-2.28] diazepam dose equivalents vs 1.60 [0.66-4.27] diazepam dose equivalents; *P* < .001) and olanzapine (median [IQR] dosage, 1.09 [0.38-2.46] mg vs 2.50 [1.16-6.65] mg; *P* < .001) (Table 3).

**Table 3.** Opiate, Benzodiazepine, and Antipsychotic Medication Administration Before Any Version of the ML-Delirium Risk Stratification Model and Following Clinical Deployment of the Fusion ML-Delirium Risk Stratification Model

Medication	Proportion receiving medication, No. (%)^a^	Dose administered per hospital d, median (IQR)	*P* value
Opiates, IV morphine dose equivalents			
Pre-ML model deployment	2462 (61.7)	4.80 (1.49-15.54)	.12
Post-ML model deployment	2003 (66.1)	3.87 (1.00-12.87)	
Benzodiazepine, diazepam dose equivalents			
Pre-ML model deployment	852 (21.3)	1.60 (0.66-4.27)	<.001
Post-ML model deployment	1352 (44.6)	0.93 (0.42-2.28)	
Haloperidol, mg			
Pre-ML model deployment	351 (8.8)	0.27 (0.10-0.64)	.12
Post-ML model deployment	498 (16.4)	0.23 (0.09-0.60)	
Quetiapine, mg			
Pre-ML model deployment	214 (5.4)	9.64 (3.70-25.49)	.13
Post-ML model deployment	370 (12.2)	8.44 (2.65-25.45)	
Olanzapine, mg			
Pre-ML model deployment	31 (0.8)	2.50 (1.17-6.65)	<.001
Post-ML model deployment	162 (5.3)	1.09 (0.38-2.46)	
Risperidone, mg			
Pre-ML model deployment	30 (0.8)	0.53 (0.23-1.83)	.75
Post-ML model deployment	43 (1.4)	0.48 (0.27-2.00)	

Abbreviations: IV, intravenous; ML, machine learning.

^a^ Includes 4068 admissions in the pre-ML period and 3031 admissions in the post-ML period.

## Discussion

In this quality improvement study, we present a fusion multimodal with NLP ML-based delirium risk stratification model developed using a vertical integration approach and demonstrated acceptable discriminative performance statistics over a 13-month period in live clinical practice. Model deployment was associated with a significant 4-fold increase in delirium detection rates. This shift in workflow allowed for focused recurrent screening of a patient population with higher risk of delirium, optimizing resource allocation. While at least 1 other ML-delirium risk stratification model with NLP tested in live clinical practice has been published,^25^ it lacked reporting on associated workflow or clinical outcomes. Similarly, 3 reports of an ML-based delirium risk stratification model without NLP tested in live clinical practice also did not report on such outcomes.^9,22,48^

We believe the observed increased hospital LOS in the post-ML cohort is attributable to a higher prevalence of delirium and a higher Elixhauser Comorbidity Index, both known factors associated with increased hospital LOS.^1,60^ Additionally, while a higher proportion of patients in the post-ML cohort received benzodiazepines, haloperidol, and olanzapine, they received significantly lower daily doses of these medications. Our service has focused on disseminating delirium prevention and treatment best practices, including psychotropic and opiate medication reduction.^51^ Given a higher Elixhauser Comorbidity Index and delirium prevalence in the post-ML cohort, it is possible that an increased prevalence of associated pain, anxiety, and agitation in this refined cohort necessitated medication use in more patients, while concomitantly, efforts were made to reduce dosing. However, this remains speculative and cannot be definitively tested with our current data.

A major strength of our model development process is the use of the highly sensitive and specific CAM delirium assessment tool^52^ by raters with proven reliability^51^ as the reference standard for the delirium diagnosis. This type of criterion standard delirium diagnostic method, which is so infrequently used in published ML-based delirium prediction models,^5,8,10,17,19,20,24,27,28,37,38,39,40,43,44,45,47,48^ distinguishes our approach from most published reports that rely solely on EMR review and *ICD-10-CM* coding.

Another strength of the model presented here is its development and validation in live clinical practice, leveraging a vertical integration pipeline.^49^ The iterative model upgrade process facilitated the resolution of practical challenges associated with model deployment. Furthermore, our model was developed using a diverse cohort of medical and surgical patients, in contrast to most published ML-based delirium predictive models that were developed and validated on more narrowly defined cohorts of medical^6,18,20,23,24,32,36,46,47^ or surgical^3,8,10,14,15,16,17,22,26,27,28,29,30,34,38,39,40,41,42,43,44,45,48^ patients. This enhances the potential generalizability of our model.

We will continue to advance our model through rigorous evaluation and clinical application. Our immediate plans involve a formal analysis of model performance at Mount Sinai Morningside Hospital in New York, New York. To ensure optimal utilization and impact, we will implement a real-time monitoring system at Mount Sinai Morningside Hospital. Additionally, we are actively exploring opportunities to expand deployment to affiliated hospitals, particularly those without dedicated delirium services. By broadening the reach of our model, we aim to improve patient care and streamline clinical workflows in diverse health care settings.

### Limitations

This study has some limitations. Any enthusiasm for this model’s generalizability should be tempered by the absence of external validation at other hospitals. Another limitation of this model’s generalizability is imposed by the fact that our model was developed to work in synergy with our unique form of multicomponent-based treatment of extant delirium, which diverges from the more common application of multicomponent-based delirium programs that focus on delirium prevention.^61^ Given that many hospital systems lack dedicated delirium programs, the model’s adoption and effective implementation would depend on the willingness of health care practitioners to adopt this delirium risk stratification tool and conduct proper patient assessments. While this does not preclude the model’s utility in such settings, further study is necessary to confirm its applicability.

## Conclusions

This quality improvement study developed and demonstrated the feasibility and clinical utility of a novel multimodal ML model to automate delirium risk stratification in live clinical practice. This model demonstrated acceptable performance in live clinical practice and may facilitate resource allocation to enhance delirium identification and care.

## Notes

Supplement 1. **eFigure 1.** Timeline for the MSH Quality Improvement Initiative **eFigure 2.** Sampling Strategy for EMR Features **eFigure 3.** Sampling Strategy for Clinical Notes: **eFigure 4.** Model Fusion Architecture of the Multimodal (+NLP) Application **eFigure 5.** Fusion Multimodal and Natural Language Processing Model Variables Ranked by Gini Importance **eTable.** Clinical And Demographic Characteristics of Fusion Model Test/Train and Fusion Model Live Clinical Deployment Validation Cohorts

Supplement 2.
Data Sharing Statement
