# Supplementary material for: Machine Learning Multimodal Model for Delirium Risk Stratification
Source: JAMA Netw Open. 2025 May 7;8(5):e258874. doi: 10.1001/jamanetworkopen.2025.8874 (PMC12059973; doi:10.1001/jamanetworkopen.2025.8874)
Supplement: Supplement 1. — eFigure 1. Timeline for the MSH Quality Improvement Initiative eFigure 2. Sampling Strategy for EMR Features eFigure 3. Sampling Strategy for Clinical Notes: eFigure 4. Model Fusion Architecture of the Multimodal (+NLP) Application eFigure 5. Fusion Multimodal and Natural Language Processing Model Variables Ranked by Gini Importance eTable. Clinical And Demographic Characteristics of Fusion Model Test/Train and Fusion Model Live Clinical Deployment Validation Cohorts [file jamanetwopen-e258874-s001.pdf]

Supplemental Online Content

Friedman JI, Parchure P, Cheng F, et al. Machine learning multimodal model for delirium risk stratification. *JAMA Netw Open*. 2025;8(5):e258874. doi:10.1001/jamanetworkopen.2025.8874

eFigure 1. Timeline for the MSH Quality Improvement Initiative

eFigure 2. Sampling Strategy for EMR Features

eFigure 3. Sampling Strategy for Clinical Notes:

eFigure 4. Model Fusion Architecture of the Multimodal (+NLP) Application

eFigure 5. Fusion Multimodal and Natural Language Processing Model Variables Ranked by Gini Importance

eTable. Clinical And Demographic Characteristics of Fusion Model Testing, Training and Fusion Model Live Clinical Deployment Validation Cohorts

This supplemental material has been provided by the authors to give readers additional information about their work.

© 2025 Friedman JI et al. *JAMA Network Open*.

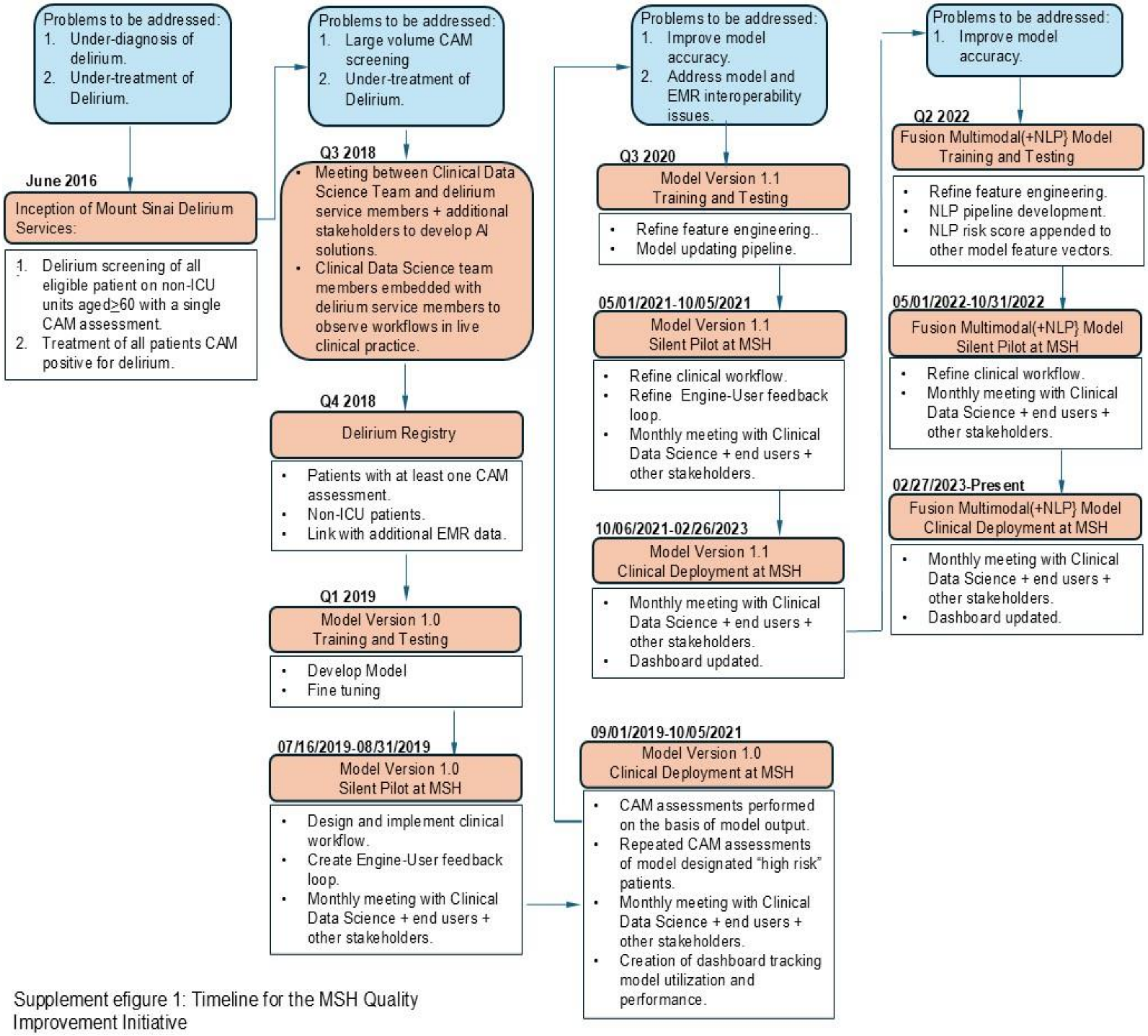

Supplement efigure 1: Timeline for the MSH Quality Improvement Initiative

© 2025 Friedman JI et al. *JAMA Network Open*.

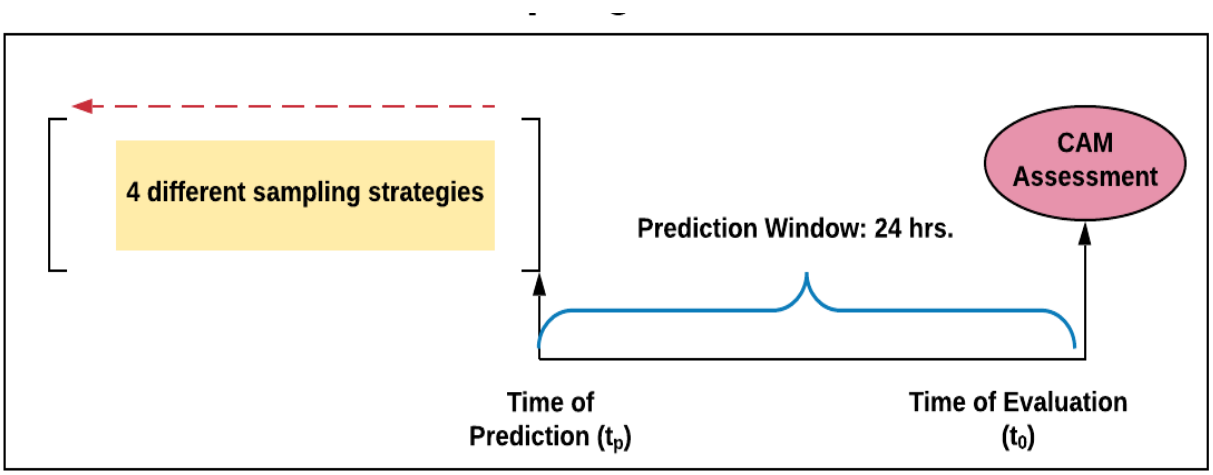

Supplement eFigure - 2 Sampling Strategy for EMR Features: To standardize model input data, a sampling module was developed to apply adaptive logic, ensuring a fixed number of observations within predefined intervals. A time series was constructed by specifying a sampling window and frequency relative to the risk stratification time (tp). The sampling window was determined based on variable availability, optimizing data completeness and minimizing missing values. The risk stratification time (tp) was set to 24 hours prior to the CAM assessment, while the sampling frequency defined the standard intervals between clinical measurements, ensuring consistency across observations.

© 2025 Friedman JI et al. *JAMA Network Open*.

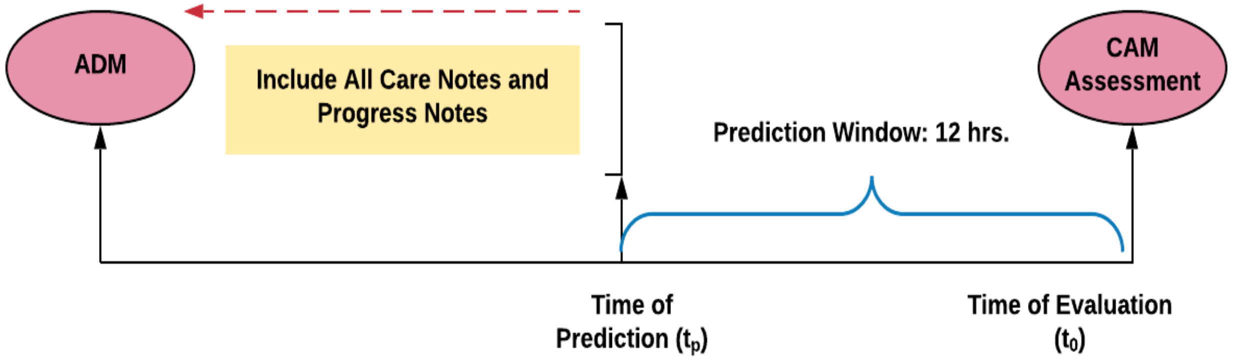

Supplement eFigure 3 - Sampling Strategy for Clinical Notes: For each inpatient admission included in the fusion model clinical notes-comprising care notes (submitted by registered nurses) and progress notes (submitted by residents, fellows, attending physicians, nurse practitioners, physician assistants)-were aggregated into a text corpus. This corpus encompassed care and progress notes sampled from the 12-hour window preceding the risk stratification time. The text corpus was processed using a sentence detection module to segment the text into individual sentences, which were then input into an NLP pipeline for tokenization, stemming, lemmatization, and the creation of 1-gram and 2-gram bag-of-words models. Term frequency rate (TFR) was calculated at the encounter level across the cohort. Words with a TFR≥0.3 in notes of delirium-positive patients were selected as candidate features. The resulting feature list was categorized into three primary categories: diagnoses, signs and symptoms, and medications. Expert clinical feedback was used to refine the feature selection, focusing on those with relevance to the presentation of delirium. These selected features were then assembled into a feature vector.

© 2025 Friedman JI et al. *JAMA Network Open*.

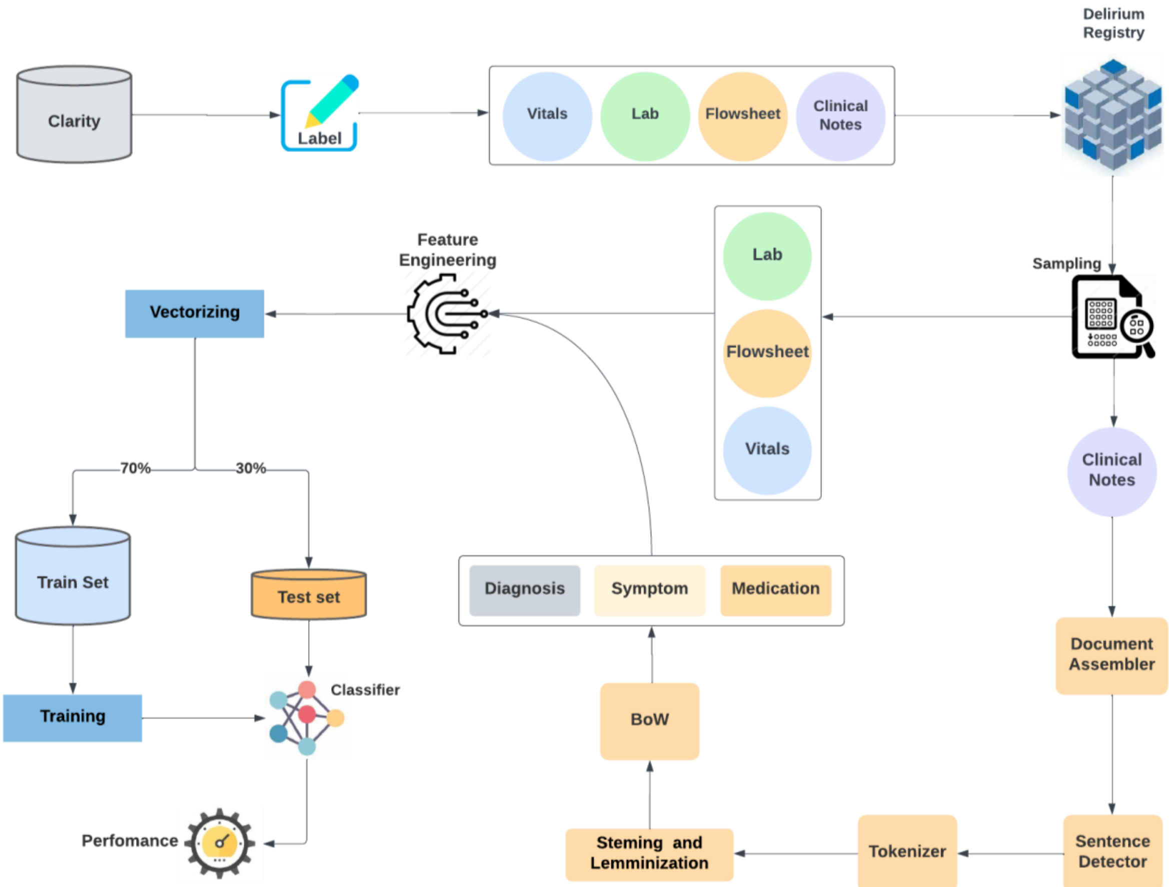

Supplement eFigure 4 - Model Fusion Architecture of the Multimodal(+NLP) Application: The under-sampled training set was utilized, incorporating feature vectors derived from both structured and semi-structured observational data. The NLP risk score was appended to these feature vectors. A 10-fold cross-validation procedure was employed to train the model using the RF algorithm. Following hyper-parameter optimization, the RFE method was applied to reduce the number of features.

© 2025 Friedman JI et al. *JAMA Network Open*.

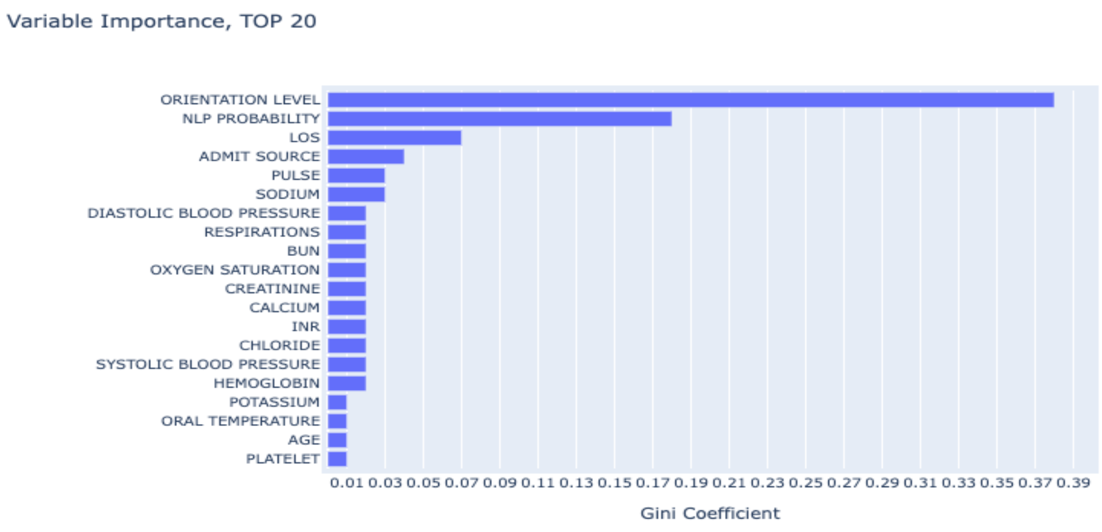

Supplement eFigure 5a: Fusion Multimodal(+NLP) model variables ranked by Gini importance:

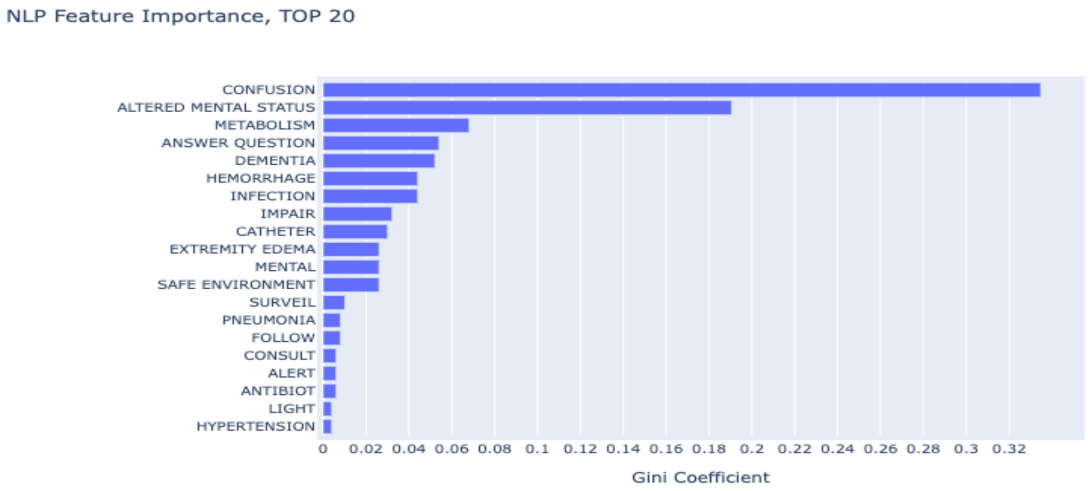

Supplement eFigure 5b: Natural Language Processing (NLP) features only model variables ranked by Gini importance:

© 2025 Friedman JI et al. *JAMA Network Open*.

Characteristic	Fusion Model Training/Testing Cohort No. (%)	Fusion Model Live Clinical Deployment Validation Cohort No. (%)
Time Period	January 1 2016 to December 31, 2020	March 1, 2023 to March 31, 2024
Number of Admissions, No.	5646	19615
Number of Unique Patients, No.	5149	14960
Number of Admissions With At Least One CAM Assessment, No.	5646	3031
Age (median [IQR]), y	73.37 [66.42-81.36]	72.11 [62.26-78.97]
**Gender**		
Women	2814 (49.8)	8894 (45.3)
Men	2792 (49.5)	10618 (54.1)
Missing*	40 (0.7)	103 (0.5)
**Race and Ethnicity**		
Asian	314 (5.6)	1337 (6.8)
Black or African American	1093 (19.4)	3730 (19.0)
Hispanic	982 (17.4)	4456 (22.7)
White	2217 (39.3)	7719 (39.4)
Other**	776 (13.7)	1875 (9.6)
Unknown	224 (4.0)	396 (2.0)
Missing	40 (0.7)	102 (0.5)
Elixhauser Comorbidity Index (median [IQR])	16.00 [4.00-29.00]	12.00 [1.00-24.00]

Supplement eTable 1: Clinical And Demographic Characteristics of Fusion Model Test/Train and Fusion Model Live Clinical Deployment Validation Cohorts.

*: Missing in post deployment cohort include one patient with gender “Indeterminate”.

**: Other contains races including American Indian or Alaska Native, Native Hawaiian or Pacific Islander, Multi-race and other.

© 2025 Friedman JI et al. *JAMA Network Open*.
