# Supplementary material for: Machine Learning Multimodal Model for Delirium Risk Stratification
Source: JAMA Netw Open. 2025 May 7;8(5):e258874. doi: 10.1001/jamanetworkopen.2025.8874 (PMC12059973; doi:10.1001/jamanetworkopen.2025.8874)
Supplement: Supplement 2. — Data Sharing Statement [file jamanetwopen-e258874-s002.pdf]

Data Sharing Statement

Friedman. Machine Learning Multimodal Model for Delirium Risk Stratification. *JAMA Netw Open*. Published May 07, 2025. doi:10.1001/jamanetworkopen.2025.8874

Data
Data available: No

Additional Information
Explanation for why data not available: Data is not available for secondary use per IRB standards.
